# The Journal of Comparative Medicine and Veterinary Archives

**Published:** 1890-01

**Authors:** 


### EDITORIAL.

---

### THE JOURNAL OF COMPARATIVE MEDICINE AND VETERINARY ARCHIVES.

*Journal.* "A periodical publication . . . giving an account of passing events, the proceedings and memoirs of societies, and the like." *Webster.*

*Archives.* "Public records and papers which are preserved as evidence of facts." *Webster.*

When, ten years ago, we inaugurated the "*Archives of Comparative Medicine and Surgery*," a quarterly Journal of the Anatomy, Pathology and Therapeutics of the lower animals, we had in view the education of a large class of medical men, whose liberal moments would tempt them from the routine of practice, on chlorotic and rich women, on anæmic and fondled children, and on maimed but exacting men, to the investigations of the healthy and morbid phenomena of other animals, whose more natural life, exemplifies more truly the principles of medicine. We knew that it would draw a large body of influential workers to the support of the handful of advanced and struggling veterinarians, who then represented the profession of the country.

In two years, aided by such writers as Berns, Brown, De Voe, Dowd, Porter, Law, Loring, Meyer, Louler, Spitzka, Heath, Hopkins, Satterthwaite, Wendt and others, we published 552 pages of matter of general interest, upon the above subjects, of which over three-fifths was composed of original communications. We found that the seed had been well sown, and that our contributors, their subjects and the demands of our readers required more than an "evidence of facts." "Passing events," and the "proceedings" of the newly organized societies of the students of our lower animals, asked for a medium through which they could make public their ideas, whether proven facts or hypothetical reasonings. Both had to be placed before the community for digestion and assimilation, and our name was changed to "*The Journal of Comparative Medicine and Surgery.*"

During the next eight years, our former contributors, with added forces, among whom are Caton, Hunt, Jennings, Mitchell, Osler, Robertson, Schmidt, Walton, Wortman, Billings, Bowler, Clement, Dana, Lindsay, Lowe, McLellen, O'Leary, Ramsdell, Walley, Walton, Johne, Miller, Salmon, Ward, Leidy, Burke, Dunham, James, Law, Peters, Rogers, Shufeldt, Sutton, Waugh, Stickler. Allen, Chapman, Duncan, Mills, Curtice, Burke, Formad, Lee, McLean, Brill, Gottheil, Marcy, Reefer and others, poured in the results of their labors to the "periodical publication" and veterinary medicine grew stiong as the result.

Instead of a couple of struggling veterinary schools and a single society with a weak and scattered profession, as existed in that day, we now find progressing veterinary colleges at all points of the compass, we find state and local veterinary societies in every community. Instead of the interested outsider being obliged to furnish the literature for our press, we have all classes of scientific men vieing to give to the public ideas for record, and we have an advanced veterinary profession, seizing the theories offered them, and analyzing the data chronicled for them, and by acute practical application making a literature of their own. The student of natural history and the veterinary practitioner find themselves more closely tied in mutual interest than ever, and, with bonds of union with the agriculturist demand a common outlet and exchange of thoughts in one publication. To meet this, we again slightly alter our title, and commence the second decade of our existance as "*The Journal of Comparative Medicine* and *Veterinary Archives.*" To furnish all matter more promptly to our increasing number of readers the *Journal* will be issued *monthly* instead of quarterly.

We will continue to note all new matters in the rapidly advancing progress made in the study of the anatomy, physiology, pathology and therapeutics of all animals, and the scientific writers who have aided in establishing the reputation of the Journal as a pioneer in publishing *original researches* in regard to our lower brethren, will continue to contribute to our pages. The monthly edition will allow us to place before our readers immediately, a complete record of all the important and interesting discoveries, news and advances in veterinary medicine. Original articles on all that pertains to our domestic animals and veterinary medicine will be followed by careful selections and translations from the best veterinary and medical journals in all languages. The proceedings of the veterinary secieties throughout the country will be carefully recorded. All the veterinary schools of the United States and Canada will be interrogated monthly for items of interest in their *personnel*, curriculum, clinical, and laboratory work. The bulletins of the Army, Department of Agriculture, and great commercial centres will be culled for transactions in the care and commerce of animals which bear upon the veterinary profession. Illustrations will be used whenever needed for the better exposition of our subjects.

The Journal will be the organ of no school, we will continue it as the medium of communication of the veterinary profession of the United States and Canada. Our pages are open to all who have at heart the well being of that profession, and the increase of our knowledge in the care of animals.

